# Care trajectory differences in women and men with end-stage renal disease after dialysis initiation

**DOI:** 10.1371/journal.pone.0289134

**Published:** 2023-09-14

**Authors:** Juliette Piveteau, Maxime Raffray, Cécile Couchoud, Valérie Chatelet, Cécile Vigneau, Sahar Bayat

**Affiliations:** 1 EHESP, CNRS, Inserm, Arènes–UMR 6051, RSMS–U1309, Univ Rennes, Rennes, France; 2 Renal Epidemiology and Information Network (REIN) Registry, Biomedecine Agency, Saint-Denis-La-Plaine, France; 3 Centre Universitaire des Maladies Rénales, CHU Caen, Caen, France; 4 U1086 Inserm, ANTICIPE, Centre de Lutte Contre le Cancer François Baclesse, Caen, France; 5 CHU Rennes, Inserm, EHESP, Irset (Institut de Recherche en Santé, Environnement et Travail)–UMR_S 1085, Univ Rennes, Rennes, France; Faculty of Medicine, Saint-Joseph University, LEBANON

## Abstract

Few studies investigated sex-related differences in care consumption after dialysis initiation. Therefore, the aim of this study was to compare the care trajectory in the first year after dialysis start between men and women by taking into account the context of dialysis initiation. All patients who started dialysis in France in 2015 were included. Clinical data of patients and context of dialysis initiation were extracted from the Renal Epidemiology and Information Network (REIN) registry. Data on care consumption in the first year after dialysis start came from the French national health data system (SNDS): hospital stays <24h, hospital stays to prepare or maintain vascular access, hospital stays >24h for kidney problems and hospital stays >24h for other problems, and consultations with a general practitioner. Variables were compared between men and women with the *χ*^2^ test and Student’s or Welch *t*-test and logistic regression models were used to identify the factors associated with care consumption after dialysis start. The analysis concerned 8,856 patients (36% of women). Men were less likely to have a hospital stays >24h for kidney problems than women (OR = 0.8, 95% CI = [0.7–0.9]) and less general practitioner consultations (OR = 0.8, 95% CI = [0.8–0.9]), in the year after dialysis initiation, after adjustment on patient’s characteristics. Moreover, hospital stays for vascular access preparation or maintenance were longer in women than men (median duration: 2 days [0–2] vs. 1 day [0–2], p < 0.001). In conclusion, despite greater comorbidities in men, this study found few differences in post-dialysis care trajectory between men and women.

## Introduction

Chronic kidney disease (CKD) is a silent disease that slowly progresses over months or years. It is defined as “*the presence of markers of kidney damage or a decrease in estimated glomerular filtration rate (eGFR) below 60ml/min/1*.*73 m^2^ for more than three months*, *regardless of its cause*” [1]. When a patient reaches end-stage renal disease (the last of the five CKD stages), kidney replacement therapy (KRT) is proposed: hemodialysis, peritoneal dialysis, or kidney transplantation. Some patients will choose conservative palliative treatments.

Disparities persist between men and women with CKD. CKD prevalence is higher in women, but paradoxically fewer women receive dialysis [2]. This prevalence variation between sexes is explained partly by the current formula to calculate eGFR that can underestimate eGFR in women, thus overestimating CKD prevalence [3]. In addition, kidney function declines more rapidly in men who have more comorbidities than in women, thus explaining the higher number of men who initiate KRT [2]. We observed also differences in the outcomes of dialysis in diabetic patients with CKD [4, 5].

Moreover, there are disparities in care consumption between men and women with CKD. Studies on post-dialysis hospitalization rates have been performed, but with discordant results. Three American studies found higher hospitalization rates in men [6–8]. Conversely, two other studies (one in the USA and one in Canada) showed that hospitalization rates are higher in women [9, 10], and this difference was particularly pronounced for younger patients [11]. Lastly, an American study showed that men are hospitalized more frequently for ischemic heart disease, and women for congestive heart failure [12]. Post-dialysis hospitalization duration also has been compared between sexes and was longer in women than men [7, 12]. Conversely, another study found no difference in terms of hospitalization rates and duration between men and women [13]. All these studies focused only on hospital stays, and none compared the use of post-dialysis outpatient care or ambulatory care by men and women. Furthermore, emergency dialysis initiation is associated with increased morbidity, mortality and hospitalization duration at dialysis start [14–17]. Yet, no study investigated the association between emergency dialysis start and post-dialysis care consumption.

Therefore, the aim of this study was to compare post-dialysis care consumption (hospital stays and ambulatory care) in men and women in France, by taking into account the context of dialysis initiation (emergency vs planned).

## Materials and methods

### Study population

The patients included in this retrospective study were adults with end-stage renal disease who initiated dialysis in France in 2015. Data were extracted from the Renal Epidemiology and Information Network (REIN) registry that collects data on all patients who initiate KRT in France [18, 19], and from the French national health data system (SNDS) that contains care consumption data.

The restrictions due to French Personal data protection regulation (CNIL) prohibit the authors from making the minimal data set publicly available. The French REIN registry that received the agreement by the CNIL (Commission Nationale de l’Information et des Libertés) in 2010 (agreement number: 903188 Version 3). All involved subjects received an information leaflet before giving their verbal consent to participate. This procedure was approved by the ethics committee.

### Data study

Two data sources were exploited in this study:

- The REIN registry to extract sociodemographic data [sex, age and activity status (active or not active)], clinical and laboratory data [body mass index (BMI), albuminemia, hemoglobin, eGFR, kidney disease and mobility (total incapacity, needs help, autonomous walking)], comorbidities [cirrhosis, active cancer, diabetes, chronic respiratory disease, behavioral disorder, and number of cardiovascular disease], first dialysis type [dialysis modality (hemodialysis, peritoneal dialysis), stand-alone dialysis (home and out-center hemodialysis, non-assisted peritoneal dialysis), dialysis initiation context (emergency or planned), and vascular access (catheter or fistula)]. The type of nephropathy were grouped in three categories: acute nephropathy, chronic nephropathy and unknown [16]. A variable combining dialysis initiation context (emergency or planned) and vascular access type (catheter or fistula) was created.
- As the REIN registry does not contain post-dialysis care consumption data, the French National Health Data System (SNDS) database was used. The SNDS contains the detailed care consumption data of 99% of the French population. Patients who initiated dialysis in 2015 and identified in the REIN registry were matched with data in the SNDS database using a previously described deterministic matching method [20, 21]. Variables extracted from the SNDS database to describe care consumption in the first year after dialysis initiation were: consultations with a general practitioner (GP), hospital stays of >24 hours for kidney-related (S1 Table) or other problems based on the International Classification of Diseases Codes, hospital stays to prepare or maintain vascular access, and hospital stays <24 hours (all diagnoses). Hospital stays for kidney-related or other problems were all hospital stays not devoted to prepare dialysis. Hospital stays for dialysis initiation were not included in these analyses.

Patients without matching between the REIN and SNDS databases, and patients without information on the context of dialysis initiation were excluded.

### Statistical analysis

The patients’ sociodemographic, clinical and laboratory characteristics, dialysis characteristics were described using numbers and percentages for categorical variables, and medians and interquartile ranges (IQR) for quantitative variables. Patients’ characteristics were compared between men and women using the *χ*^2^ test for categorical variables and the Student’s or Welch *t*-test for quantitative variables.

Patients were followed until death, kidney transplantation, or for one year after dialysis initiation. The post dialysis care consumption (per person-year) in the year after dialysis initiation were calculated and compared between men and women: GP consultations, hospital stays of >24 hours for kidney-related problems, hospital stays of >24 hours for other problems, hospital stays to prepare or maintain vascular access, and hospital stays <24 hours. The number of GP consultations and hospital stays by person-year, and the duration of hospital stays have been compared by sex using the Student’s or Welch t-test. We have also compared the characteristic of the first and second hospitalization between women and men using the Student’s test. Finally, we also used logistic regression models to identify the factors associated with hospital stays or GP consultation after dialysis initiation. The models have been adjusted on patients’ sociodemographic and clinical characteristics, and care consumption before dialysis. Statistical analyses were done with R 4.0.2 and comparisons were considered significant when p-value <5%.

## Results

### Description of the study population at dialysis initiation

In 2015, 10,667 patients started dialysis in France, and 8,856 of them were included in this study after exclusion of patients without matching between the REIN and SNDS databases (90% could be matched) and without data on dialysis initiation context [21].

The mean age of the included patients was 68 years, and 36% were women. Thirteen percent of patients have died in the year after dialysis initiation. Comparison of the sociodemographic, clinical and laboratory characteristics (Table 1) between sexes showed that men were more frequently smokers and former smokers, but they were more active and had better mobility than women. At dialysis initiation, men had more comorbidities (chronic respiratory disease, cirrhosis, active cancer, and cardiovascular disease), whereas women had more behavioral disorders (Table 2). The rate of dialysis initiation in emergency was comparable between sexes. Conversely, in the planned dialysis initiation group, men started more often dialysis with a fistula (Table 2). Similar between-sex differences were observed when patients were divided in two groups in function of the dialysis initiation context (in emergency and planned manner) (S2 Table).

**Table 1:** Sociodemographic, clinical and laboratory characteristics of women and men who initiated dialysis in France, in 2015 (N = 8,856).

	Women	Men	Total Number (%)	p-value (Chi^2^ test)
	N = 3161	N = 5695		
	Number (%)	Number (%)		
**Age (years)**				**0.03**
**18–45**	269 (8%)	467 (8%)	736 (8%)	
**45–60**	523 (17%)	865 (15%)	1388 (16%)	
**60–75**	1064 (34%)	2094 (37%)	3158 (36%)	
**> 75**	1305 (41%)	2269 (40%)	3574 (40%)	
**BMI (kg/m^2^)**				**< 0.001**
**< 18.5**	123 (4%)	120 (2%)	243 (3%)	
**18.5–23**	622 (20%)	1170 (21%)	1792 (20%)	
**23–25**	341 (11%)	811 (14%)	1152 (13%)	
**25–30**	718 (23%)	1651 (29%)	2369 (27%)	
**≥ 30**	807 (25%)	1012 (18%)	1819 (20%)	
**Missing**	550 (17%)	931 (16%)	1481 (17%)	
**Employment**				**< 0.001**
**Not active**	2575 (91%)	4541 (88%)	7116 (89%)	
**Active**	243 (9%)	614 (12%)	857 (11%)	
**Tobacco**				**< 0.001**
**Smoker/Ex-smoker**	536 (17%)	2670 (47%)	3206 (36%)	
**No-smoker**	2091 (66%)	2096 (37%)	4187 (47%)	
**Missing**	534 (17%)	929 (16%)	1463 (17%)	
**Nephropathy type**				**< 0.001**
**Acute**	435 (14%)	641 (11%)	1076 (12%)	
**Chronic**	2063 (65%)	3900 (68%)	5963 (67%)	
**Unknown**	663 (21%)	1154 (20%)	1817 (21%)	
**Albuminemia (g/L)**				**0.2**
**< 30**	570 (18%)	987 (17%)	1557 (18%)	
**≥ 30**	2144 (68%)	3985 (70%)	6129 (69%)	
**Missing**	447 (14%)	723 (13%)	1170 (13%)	
**eGFR (mL/min/1.73 m** ^**2**^ **)**				**< 0.001**
**5–9**	1615 (51%)	2701 (47%)	4316 (49%)	
**< 5**	482 (15%)	734 (13%)	1216 (14%)	
**10–14**	527 (17%)	1254 (22%)	1781 (20%)	
**15–19**	128 (4%)	246 (4%)	374 (4%)	
**≥ 20**	88 (3%)	214 (4%)	302 (3%)	
**Missing**	321 (10%)	546 (10%)	867 (10%)	
**Hemoglobin (g/dL)**				**< 0.001**
**< 10**	1786 (57%)	2974 (52%)	4760 (54%)	
**10–11**	899 (28%)	1698 (30%)	2597 (29%)	
**≥ 12**	350 (11%)	818 (14%)	1168 (13%)	
**Missing**	126 (4%)	205 (4%)	331 (4%)	
**Mobility**				**< 0.001**
**Total incapacity**	154 (5%)	225 (4%)	379 (4%)	
**Needs help**	402 (13%)	561 (10%)	963 (11%)	
**Autonomous walking**	2358 (74%)	4438 (78%)	6796 (77%)	
**Missing**	247 (8%)	471 (8%)	718 (8%)	

BMI, Body Mass Index; eGFR, estimated glomerular filtration rate; Values are expressed as N (%); for the between-sex comparison (Chi^2^ test) missing values were not considered.

**Table 2:** Comorbidities and dialysis characteristics in women and men who initiated dialysis in France, in 2015 (N = 8,856).

Variable	Women	Men	Total Number (%)	p-value (Chi^2^ test)
	N = 3161	N = 5695		
	Number (%)	Number (%)		
**Cirrhosis**				**< 0.001**
**No**	3029 (96%)	5367 (94%)	8396 (95%)	
**Yes**	51 (2%)	179 (3%)	64 (1%)	
**Missing**	81 (2%)	149 (3%)	230 (4%)	
**Active cancer**				**< 0.001**
**No**	2796 (89%)	4899 (86%)	7695 (87%)	
**Yes**	289 (9%)	653 (11%)	942 (11%)	
**Missing**	76 (2%)	143 (3%)	219 (2%)	
**Diabetes**				0.3
**No**	1746 (55%)	3084 (54%)	4830 (54%)	
**Yes**	1401 (44%)	2585 (45%)	3986 (45%)	
**Missing**	14 (1%)	26 (1%)	40 (%)	
**Chronic respiratory disease**				**< 0.001**
**No**	2694 (85%)	4457 (78%)	7151 (81%)	
**Yes**	366 (12%)	1046 (18%)	1412 (16%)	
**Missing**	101 (3%)	192 (4%)	293 (3%)	
**Number of cardiovascular diseases**				**< 0.001**
**0**	1671 (53%)	2231 (39%)	3902 (44%)	
**1**	715 (23%)	1295 (23%)	2010 (23%)	
**2**	438 (14%)	946 (17%)	1384 (16%)	
**≥ 3**	337 (11%)	1223 (21%)	1560 (18%)	
**Disability**				0.9
**No**	2965 (94%)	5336 (94%)	8301 (94%)	
**Yes**	196 (6%)	359 (6%)	555 (6%)	
**Behavioral disorder**				**0.04**
**No**	2794 (88%)	5088 (89%)	7882 (89%)	
**Yes**	111 (4%)	155 (3%)	266 (3%)	
**Missing**	256 (8%)	452 (8%)	708 (8%)	
**Treatment**				0.3
**Peritoneal dialysis**	305 (10%)	507 (9%)	812 (9%)	
**Hemodialysis**	2856 (90%)	5188 (91%)	8044 (91%)	
**Stand-alone dialysis**				0.4
**No**	2868 (91%)	5129 (90%)	7997 (91%)	
**Yes**	287 (9%)	548 (10%)	835 (9%)	
**Missing**	6 (0%)	18 (0%)	24 (0%)	
**Dialysis initiation and vascular access**				**<0.001**
**Planned with fistula**	1208 (38%)	2306 (40%)	3514 (40%)	
**Planned with catheter**	857 (27%)	1349 (24%)	2206 (25%)	
**Emergency with fistula**	115 (4%)	263 (5%)	378 (4%)	
**Emergency with catheter**	785 (25%)	1470 (26%)	2255 (25%)	
**Missing**	196 (6%)	307 (5%)	503 (6%)	

Values are expressed as N (%); for the between-sex comparison (Chi^2^ test) missing values were not considered.

### Comparison of post-dialysis care consumption by sex

Among the 8,856 patients included in the study, 7,899 patients had at least one hospital stay in the year after dialysis initiation. Among the 42,106 hospital stays extracted from the SNDS database for these patients, 34% concerned women. Overall, 51% were hospital stays <24h, 4% were hospital stays >24h for kidney problems, 38% were hospital stays >24h for kidney-unrelated problems, and 11% were hospital stays to prepare or maintain the vascular access.

In the first year after dialysis initiation, women had fewer hospital stays <24h and hospital stays >24h for kidney-unrelated problems compared with men (S3 Table). Comparison of the number of consultations with a GP and hospital stays (any type) per person-year did not highlight any difference between sexes (Table 3).

**Table 3:** Mean number of consultations and hospital stays by person-year in men and women (N = 8,856).

	Women	Men	p-value (Welch t-test)
	N = 3161	N = 5695	
	Mean ± sd	Mean ± sd	
**Consultations with a GP**	7.5 ± 17.3	7.2 ± 23.4	0.4
**Hospital stays >24h for kidney problems**	0.4 ± 2.8	0.4 ± 9.8	>0.9
**Hospital stays >24h for other problem**	2.8 ± 9.5	3 ± 15.9	0.4
**Hospital stays to prepare for dialysis**	0.8 ± 6.9	0.7 ± 3.1	0.4
**Hospital stays <24h**	3.8 ± 19.5	4 ± 25.3	0.6

Sd: standard deviation

We used logistic regression models to study the association between sex and care consumption after dialysis start. These models were adjusted on patients’ characteristics and care consumption before dialysis (S4–S8 Tables). We found no significant association between sex and the number of hospital stays for other problems, hospital stays to prepare or maintain the vascular access, ambulatory stays. However, men had less risk to be hospitalized for kidney problem (OR = 0.8, 95% CI = [0.7; 0.9]) and to consult a GP more than 7 times (OR = 0.8 95% CI = [0.8; 0.9]) in the year after dialysis initiation. Patients who started dialysis in emergency with a catheter had more risk to be hospitalized for kidney problem in the year after dialysis initiation than patients who started a planned dialysis with fistula. In addition, the patients who started dialysis in emergency (with a catheter or fistula) and those started a planned dialysis with catheter had more risk to be hospitalized for kidney-unrelated problem more than 3 times in the year after dialysis initiation than those who started planned dialysis with fistula.

The median hospitalization length (excluding stays < 24h) was 3 days (IQR = 2–7) and each patient had, on average, five hospital stays (IQR = 2–3). Hospital stays were longer for older patients. No difference in hospital stay length was observed between men and women (p = 0.34).

Hospital stays to prepare or maintain vascular access were longer in women than men: average time of 2 days ± 3 and 1 day ± 3, respectively (p < 0.001). However, no difference in hospital stay duration (all causes combined) was found (Table 4).

**Table 4:** Duration of hospital stays by sex (N = 42,106).

	Women	Men	p-value (Welsh *t*-test)
	N = 14299	N = 27807	
	Mean ± sd	Mean ± sd	
**Duration of hospital stays >24h for kidney problems (days)**	10 ± 11	9 ± 10	0.2
**Duration of hospital stays >24h for other problems (days)**	7 ± 10	7 ± 10	0.05
**Duration of hospital stays to prepare or maintain a vascular access (day)**	2 ± 3	1 ± 3	**< 0.001**

IQR: interquartile range

Patients were hospitalized in 1 to 8 different hospitals, and 54% of patients in more than one hospital. No difference between sexes was found in the number of hospitals. Men were more often hospitalized in public-sector hospitals, whereas women had more hospital stays in private for profit clinics (S9 Table). The main reasons for hospitalization (main diagnosis code) were chemotherapy (15%), preparation or maintenance of the vascular access (12%), and control exams for treatments related to other pathologies (4%). No difference in main diagnosis codes was found between sexes.

### Description of the first and second hospitalization

In the first year after dialysis initiation, 7,899 patients were hospitalized at least once and among them 6,636 patients (84%) had a second hospital stay. The median interval between dialysis initiation and first hospitalization was 43 days [IQR = 17–97], and the median interval between first and second hospitalization was 36 days [IQR = 12–85]. No significant sex-related difference was found concerning hospital stay duration (2 days) and interval between dialysis initiation and hospitalization and between hospitalizations (Table 5).

**Table 5:** Characteristics of first and second hospitalization in men and women in the first year after dialysis initiation.

	First hospitalization (N = 7899) Mean ± sd				Second hospitalization (N = 6636) Mean ± sd		
	Women (N = 2802)	Men (N = 5097)	p-value	Women (N = 2331)		Men (N = 4305)	p-value
**Duration of hospital stay (days)**	4 ± 8	4 ± 8	>0.9	4 ± 8		4 ± 8	0.4
**Interval between dialysis start and hospitalization (days)**	74 ± 82	71 ± 77	0.15	122 ± 93		119 ± 92	0.14
**Interval between first and second hospitalization (days)**				61 ± 66		58 ± 64	0.08

IQR: interquartile range

Among the 7,899 patients with a first hospitalization in the first year after dialysis initiation, 38% were hospitalized again in the 30 days after their first hospitalization. These patients had more frequently started dialysis in emergency with a catheter (Table 6). The hospital types and reasons of hospitalization (main diagnosis code) were similar for the first and second hospitalization.

**Table 6:** Comparison of dialysis initiation contexts in patients who had at least two hospital stays after dialysis initiation in function of the interval between first and second hospital stay (N = 6,636).

	Not readmitted within 30 days	Readmitted within 30 days	p-value (Chi^2^ test)
	N = 3611	N = 3025	
	Number (%)	Number (%)	
**Condition of dialysis initiation**			**< 0.001**
**Planned dialysis with fistula**	1325 (39%)	957 (34%)	
**Planned dialysis with catheter**	967 (28%)	834 (29%)	
**Emergency dialysis with fistula**	153 (5%)	114 (4%)	
**Emergency dialysis with catheter**	956 (28%)	930 (33%)	

## Discussion

This French study is the first to compare entire post-dialysis care consumption (hospital stays and ambulatory care) by men and women who started dialysis in France in 2015. Overall, women had fewer hospital stays <24h and hospital stays >24h for kidney-unrelated problems compared with men. When care consumption was calculated per person-year, the number of consultations with a GP and the number of hospitalizations, regardless of the type of stays, were comparable between sexes, although men had more comorbidities. The multivariate analyses, adjusted on patients’ characteristics, showed that men had less hospital stays > 24h for kidney problem and less GP consultations in the year after dialysis. Post-dialysis hospital stay duration to prepare or maintain a vascular access was longer in women than men. Similar results were obtained when patients were divided in two subgroups in function of the dialysis initiation context (in emergency or planned).

Previous studies did not analyze post-dialysis GP consultation frequency in men and women and when they studied hospitalizations after dialysis initiation did not take into account the different types of hospital stays (i.e., for kidney-related and -unrelated causes) and their duration [7, 12, 13]. As mentioned in the Introduction, these studies reported contradictory results on hospitalization rate differences between sexes (higher in men, higher in women, no difference). Moreover, only two of all these studies were carried out after 2010 [10, 11]. As end-stage renal disease management and patient profiles have changed in the last decade, it is difficult to compare our results with older studies. Moreover, the two studies performed after 2010 focused only on patients receiving peritoneal dialysis [10] and only on patients receiving hemodialysis [11]. The present study included all incident patients who started dialysis (hemodialysis or peritoneal dialysis) in France in 2015.

Our study highlighted few differences in post-dialysis care consumption in men and women. Men had more hospital stays <24h and hospital stays >24h for kidney-unrelated problems. However, after taking into account patients’ age and comorbidities men had less hospital stays > 24h for kidney problem and less GP consultations. These results could be explained partly by the fact that cardiovascular and respiratory diseases and cirrhosis, which were more frequent in men, often require strict monitoring by a doctor, mainly specialists like cardiologists, to prevent acute decompensation and hospitalizations. These comorbidities can be easily controlled once dialysis has started. Conversely, women had more behavioral disorders and were less autonomous (more undernutrition, more incapacity to walk). These comorbidities cannot be controlled by starting dialysis, and this may explain the need of more frequent GP consultations. Moreover, reduced autonomy is a factor that may extend hospital stay duration.

One of the principal strengths of this study is the use of data from the SNDS database and REIN registry. This allowed obtaining clinical and laboratory data at dialysis initiation and also information on the post-dialysis care trajectory, particularly consultations with a GP and hospital stays (causes and duration). Moreover, almost all patients who started dialysis in France in 2015 could be included.

One limitation of this study is the absence of information on the patients’ socioeconomic status (e.g., socio-professional category). Furthermore, differences in care trajectory were not analyzed at a finer geographical scale (e.g., region).

Overall, women had more GP consultations and hospital stays >24h for kidney problem after adjustment on patient’s characteristics. The last difference was the duration of hospital stay to prepare or maintain a vascular access was longer and more difficult in women, possibly because vessels are smaller in women.

## Supporting information

S1 Table Diagnostic codes of hospital stays of >24 hours for kidney-related. (DOCX)

S2 Table Sociodemographic characteristics of women and men who initiated dialysis in France, in 2015 (N = 8,856). (DOCX)

S3 Table Clinical and laboratory characteristics of women and men who initiated dialysis in France, in 2015 (N = 8,856). (DOCX)

S4 Table Comorbidities in women and men who initiated dialysis in France, in 2015 (N = 8,856). (DOCX)

S5 Table Dialysis characteristics of women and men who initiated dialysis in France, in 2015 (N = 8,856). (DOCX)

S6 Table Sociodemographic, clinical, laboratory characteristics, comorbidities, dialysis characteristics of patients who initiated dialysis in France, in 2015 (by condition of dialysis initiation and sex) (N = 8,856). (DOCX)

S7 Table Comparison of care consumption in men and women in the year after dialysis initiation (N = 8,856 patients). (DOCX)

S8 Table Number of hospital stays for men and women by hospital type (N = 42,106). (DOCX)

S9 Table Comparison of care consumption in patients who had at least two hospital stays according to the interval between the first and second stay (N = 6,636). (DOCX)
