# Supplementary material for: Care trajectory differences in women and men with end-stage renal disease after dialysis initiation
Source: PLoS One. 2023 Sep 14;18(9):e0289134. doi: 10.1371/journal.pone.0289134 (PMC10501619; doi:10.1371/journal.pone.0289134)
Supplement: S1 Table — (DOCX) [file pone.0289134.s001.docx]

## **S1 Table: Diagnostic codes of hospital stays of >24 hours for kidney-related**

| Diagnostic codes | | | | |
| --- | --- | --- | --- | --- |
| N185 |  | **N12** |  | **N041** |
| N10 |  | **N138** |  | **N084** |
| N178 |  | **N139** |  | **N135** |
| N179 |  | **N142** |  | **N141** |
| N189 |  | **N159** |  | **N0210** |
| N170 |  | **N171** |  | **N085** |
| N184 |  | **N172** |  | **N119** |
| N131 |  | **N258** |  | **N0330** |
| N136 |  | **N26** |  | **N0380** |
| N200 |  | **N002** |  | **N0400** |
| N281 |  | **N004** |  | **N042** |
| N288 |  | **N007** |  | **N118** |
| N083 |  | **N015** |  | **N081** |
| N111 |  | **N0179** |  | **N132** |
| N151 |  | **N025** |  | **N137** |
| N280 |  | **N028** |  | **N160** |
| N133 |  | **N032** |  | **N163** |
| N183 |  | **N0389** |  | **N165** |
| N19 |  | **N039** |  | **N181** |
| N110 |  | **N045** |  | **N202** |
| N182 |  | **N048** |  | **N209** |
| N23 |  | **N049** |  | **N210** |
| N130 |  | **N058** |  | **N270** |
| N201 |  | **N059** |  | **N291** |
| N029 |  | **N061** |  |  |
