# Supplementary material for: Care trajectory differences in women and men with end-stage renal disease after dialysis initiation
Source: PLoS One. 2023 Sep 14;18(9):e0289134. doi: 10.1371/journal.pone.0289134 (PMC10501619; doi:10.1371/journal.pone.0289134)
Supplement: S2 Table — (DOCX) [file pone.0289134.s002.docx]

## **S2 Table: Sociodemographic, clinical, laboratory characteristics, comorbidities, dialysis characteristics of patients who initiated dialysis in France, in 2015 (by condition of dialysis initiation and sex) (N=8,856).**

|  | **Planned dialysis** | | | | **Emergency dialysis** | | | |
| --- | --- | --- | --- | --- | --- | --- | --- | --- |
|  | **Women**  **N=2242**  **Number (%)** | **Men**  **N=3933**  **Number (%)** | **Total**  **N=6175**  **Number (%)** | **p-value**  **(Chi^2^ test)** | **Women**  **N=919**  **Number (%)** | **Men**  **N=1762**  **Number (%)** | **Total**  **N=2681**  **Number (%)** | **p-value (Chi^2^ test)** |
| **Sociodemographic characteristics** | |  |  |  |  |  |  |  |
| **Age (years)** |  |  |  | 0.11 |  |  |  | 0.14 |
| **18 – 45** | 187 (8%) | 299 (8%) | 486 (7%) |  | 82 (9%) | 168 (9%) | 250 (9%) |  |
| **45 – 60** | 380 (17%) | 608 (15%) | 988 (16%) |  | 143 (16%) | 257 (15%) | 400 (15%) |  |
| **60 – 75** | 760 (34%) | 1439 (37%) | 2199 (36%) |  | 304 (33%) | 655 (37%) | 959 (36%) |  |
| **> 75** | 915 (41%) | 1587 (40%) | 2502 (41%) |  | 390 (42%) | 682 (39%) | 1072 (40%) |  |
| **BMI (kg/m²)** |  |  |  | **< 0.001** |  |  |  | **< 0.001** |
| **< 18.5** | 78 (3%) | 70 (2%) | 148 (2%) |  | 45 (5%) | 50 (3%) | 95 (3%) |  |
| **18.5 – 23** | 440 (20%) | 738 (19%) | 1178 (19%) |  | 182 (20%) | 432 (25%) | 614 (23%) |  |
| **23 – 25** | 248 (11%) | 587 (15%) | 835 (14%) |  | 93 (10%) | 224 (12%) | 317 (12%) |  |
| **25 – 30** | 517 (23%) | 1218 (31%) | 1735 (28%) |  | 201 (22%) | 433 (25%) | 634 (24%) |  |
| **≥ 30** | 606 (27%) | 734 (19%) | 1340 (22%) |  | 201 (22%) | 278 (16%) | 479 (18%) |  |
| **Missing** | 353 (16%) | 586 (14%) | 939 (15%) |  | 197 (21%) | 345 (19%) | 542 (20%) |  |
| **Activity** |  |  |  | **0.002** |  |  |  | **< 0.001** |
| **Not active** | 1808 (81%) | 3143 (80%) | 4951 (80%) |  | 767 (84%) | 1398 (79%) | 2165 (81%) |  |
| **Active** | 184 (8%) | 427 (11%) | 611 (10%) |  | 59 (6%) | 187 (11%) | 246 (9%) |  |
| **Missing** | 250 (11%) | 363 (9%) | 613 (10%) |  | 93 (10%) | 177 (10%) | 270 (10%) |  |
| **Tobacco** |  |  |  | **< 0.001** |  |  |  | **< 0.001** |
| **Smoker/ex-smoker** | 381 (17%) | 1827 (46%) | 2208 (36%) |  | 155 (17%) | 843 (48%) | 998 (37%) |  |
| **Non-smoker** | 1484 (66%) | 1458 (37%) | 2942 (48%) |  | 607 (66%) | 638 (36%) | 1245 (47%) |  |
| **Missing** | 377 (17%) | 648 (17%) | 1025 (16%) |  | 157 (17%) | 281 (16%) | 438 (16%) |  |
| **Clinical and laboratory characteristics** | |  |  |  |  |  |  |  |
| **Type of nephropathy** |  |  |  | **0.03** |  |  |  | **0.009** |
| **Acute** | 266 (12%) | 387 (10%) | 653 (11%) |  | 169 (18%) | 254 (14%) | 423 (16%) |  |
| **Chronic** | 1535 (68%) | 2792 (71%) | 4327 (70%) |  | 528 (57%) | 1108 (63%) | 1636 (61%) |  |
| **Unknown** | 441 (20%) | 754 (19%) | 1195 (19%) |  | 222 (24%) | 400 (23%) | 622 (23%) |  |
| **Albuminemia (g/L)** |  |  |  | 0.3 |  |  |  | 0.3 |
| **< 30** | 324 (14%) | 542 (14%) | 866 (14%) |  | 246 (27%) | 445 (25%) | 691 (26%) |  |
| **≥ 30** | 1614 (72%) | 2924 (74%) | 4538 (74%) |  | 530 (58%) | 1061 (60%) | 1591 (59%) |  |
| **Missing** | 304 (14%) | 467 (12%) | 771 (12%) |  | 143 (15%) | 256 (15%) | 399 (15%) |  |
| **eGFR (mL/min/1.73 m^2^)** |  |  |  | **< 0.001** |  |  |  | **0.04** |
| **5 – 9** | 1219 (54%) | 1976 (50%) | 3195 (52%) |  | 214 (23%) | 363 (21%) | 577 (21%) |  |
| **< 5** | 268 (12%) | 371 (10%) | 639 (10%) |  | 396 (43%) | 725 (41%) | 1121 (42%) |  |
| **10 – 14** | 402 (18%) | 946 (24%) | 1348 (22%) |  | 125 (14%) | 308 (17%) | 433 (16%) |  |
| **15 – 19** | 93 (4%) | 162 (4%) | 255 (4%) |  | 35 (4%) | 84 (5%) | 119 (5%) |  |
| **≥ 20** | 47 (2%) | 125 (3%) | 172 (3%) |  | 41 (4%) | 89 (5%) | 130 (5%) |  |
| **Missing** | 213 (10%) | 353 (9%) | 566 (9%) |  | 108 (12%) | 193 (11%) | 301 (11%) |  |
| **Hemoglobin (g/dL)** |  |  |  | **< 0.001** |  |  |  | **0.03** |
| **< 10** | 1167 (52%) | 1851 (47%) | 3018 (49%) |  | 619 (67%) | 1123 (64%) | 1742 (65%) |  |
| **10 – 11** | 708 (32%) | 1304 (33%) | 2012 (33%) |  | 191 (21%) | 394 (22%) | 585 (22%) |  |
| **≥ 12** | 287 (13%) | 652 (17%) | 939 (15%) |  | 63 (7%) | 166 (9%) | 229 (8%) |  |
| **Missing** | 80 (3%) | 126 (3%) | 206 (3%) |  | 46 (5%) | 79 (5%) | 125 (5%) |  |
| **Mobility** |  |  |  | **< 0.001** |  |  |  | **0.03** |
| **Total incapacity** | 79 (4%) | 114 (3%) | 193 (3%) |  | 75 (8%) | 111 (6%) | 186 (7%) |  |
| **Needs help** | 254 (11%) | 317 (8%) | 571 (9%) |  | 148 (16%) | 244 (14%) | 392 (15%) |  |
| **Autonomous walking** | 1754 (78%) | 3202 (81%) | 4956 (80%) |  | 604 (66%) | 1236 (70%) | 1840 (68%) |  |
| **Missing** | 155 (7%) | 300 (8%) | 455 (8%) |  | 92 (10%) | 171 (10%) | 263 (10%) |  |
| **Comorbidities** |  |  |  |  |  |  |  |  |
| **Cirrhosis** |  |  |  | **< 0.001** |  |  |  | 0.06 |
| **No** | 2158 (96%) | 3717 (95%) | 5875 (94%) |  | 871 (95%) | 1650 (94%) | 2521 (94%) |  |
| **Yes** | 32 (1%) | 119 (3%) | 151 (3%) |  | 19 (2%) | 60 (3%) | 79 (3%) |  |
| **Missing** | 52 (2%) | 97 (2%) | 149 (3%) |  | 29 (3%) | 52 (3%) | 81 (3%) |  |
| **Active cancer** |  |  |  | **< 0.001** |  |  |  | 0.6 |
| **No** | 2022 (90%) | 3434 (87%) | 5456 (88%) |  | 774 (84%) | 1465 (83%) | 2239 (83%) |  |
| **Yes** | 170 (8%) | 413 (11%) | 583 (10%) |  | 119 (13%) | 240 (14%) | 359 (13%) |  |
| **Missing** | 50 (2%) | 86 (2%) | 136 (2%) |  | 26 (3%) | 57 (3%) | 83 (4%) |  |
| **Diabetes** |  |  |  | 0.4 |  |  |  | 0.7 |
| **No** | 1257 (56%) | 2162 (55%) | 3419 (55%) |  | 489 (53%) | 922 (52%) | 1411 (53%) |  |
| **Yes** | 974 (43%) | 1753 (44%) | 2727 (44%) |  | 427 (46%) | 832 (47%) | 1259 (47%) |  |
| **Missing** | 11 (1%) | 18 (1%) | 29 (1%) |  | 3 (1%) | 8 (1%) | 11 (0.4%) |  |
| **Chronic respiratory disease** |  |  |  | **< 0.001** |  |  |  | **< 0.001** |
| **No** | 1928 (86%) | 3143 (80%) | 5069 (82%) |  | 766 (83%) | 1316 (75%) | 2082 (78%) |  |
| **Yes** | 246 (11%) | 668 (17%) | 914 (15%) |  | 120 (13%) | 378 (21%) | 498 (18%) |  |
| **Missing** | 68 (3%) | 124 (3%) | 192 (3%) |  | 33 (3%) | 68 (4%) | 101 (4%) |  |
| **Number of cardiovascular diseases** |  |  |  | **< 0.001** |  |  |  | **< 0.001** |
| **0** | 1261 (56%) | 1627 (41%) | 2888 (47%) |  | 410 (45%) | 604 (34%) | 1014 (38%) |  |
| **1** | 491 (22%) | 921 (23%) | 1412 (23%) |  | 224 (24%) | 374 (21%) | 598 (22%) |  |
| **2** | 292 (13%) | 623 (16%) | 915 (15%) |  | 146 (16%) | 323 (18%) | 469 (17%) |  |
| **≥ 3** | 198 (9%) | 762 (19%) | 960 (16%) |  | 139 (15%) | 461 (26%) | 600 (22%) |  |
| **Disability** |  |  |  | 0.5 |  |  |  | 0.2 |
| **No** | 2100 (94%) | 3703 (94%) | 5803 (94%) |  | 865 (94%) | 1633 (93%) | 2498 (93%) |  |
| **Yes** | 142 (6%) | 230 (6%) | 372 (6%) |  | 54 (6%) | 129 (7%) | 183 (7%° |  |
| **Behavioral disorder** |  |  |  | **0.009** |  |  |  | **0.9** |
| **No** | 1999 (90%) | 3553 (90%) | 5552 (90%) |  | 795 (86%) | 1535 (87%) | 2330 (87%) |  |
| **Yes** | 84 (4%) | 100 (3%) | 184 (3%) |  | 27 53%) | 55 (3%) | 82 (3%) |  |
| **Missing** | 159 (7%) | 280 (7%) | 439 (7%) |  | 97 (11%) | 172 (10%) | 269 (10%) |  |
| **Dialysis characteristics** | |  |  |  |  |  |  |  |
| **Stand-alone dialysis** |  |  |  | 0.4 |  |  |  | 0.4 |
| **No** | 1965 (88%) | 3409 (87%) | 5374 (87%) |  | 903 (98%) | 1720 (98%) | 2623 (98%) |  |
| **Yes** | 271 (12%) | 507 (13%) | 778 (13%) |  | 16 (2%) | 41 (2%) | 57 (2%) |  |
| **Missing** | 6 (0.3%) | 17 (0.4) | 23 (0.3) |  | 0 | 1 (0%) | 1 (0%) |  |
| **Vascular access** | |  |  | **< 0.001** |  |  |  | 0.11 |
| **Fistula** | 1208 (54%) | 2306 (59%) | 3514 (57%) |  | 115 (13%) | 263 (15%) | 378 (14%) |  |
| **Catheter** | 857 (38%) | 1349 (34%) | 2206 (36%) |  | 785 (85%) | 1470 (83%) | 2255 (84%) |  |
| **Missing** | 177 (8%) | 278 (7%) | 455 (7%) |  | 19 (2%) | 29 (2%) | 48 (2%) |  |

Values are expressed as N (%); for the between-sex comparison (Chi^2^ test) missing values were not considered
