# Supplementary material for: Care trajectory differences in women and men with end-stage renal disease after dialysis initiation
Source: PLoS One. 2023 Sep 14;18(9):e0289134. doi: 10.1371/journal.pone.0289134 (PMC10501619; doi:10.1371/journal.pone.0289134)
Supplement: S3 Table — (DOCX) [file pone.0289134.s003.docx]

## **S3 Table. Comparison of care consumption in men and women in the year after dialysis initiation (N=8,856 patients)**

|  | **Women**  **N=3161**  **Number (%)** | **Men**  **N=5695**  **Number (%)** | **Total**  **Number (%)** | **p-value (Chi2 test)** |
| --- | --- | --- | --- | --- |
| **Consultation with a GP in the year after dialysis initiation** |  |  |  | 0.08 |
| **0** | 550 (17%) | 1051 (18%) | 1601 (18%) |  |
| **[1-7]** | 1732 (55%) | 3183 (56%) | 4915 (55%) |  |
| **≥ 8** | 879 (28%) | 1461 (26%) | 2340 (26%) |  |
| **Hospital stays >24h for kidney problems in the year after dialysis initiation** |  |  |  | 0.3 |
| **0** | 2760 (87%) | 4991 (88%) | 7751 (87%) |  |
| **1** | 297 (9%) | 548 (10%) | 845 (9%) |  |
| **≥ 2** | 104 (3%) | 156 (3%) | 260 (3%) |  |
| **Hospital stays >24h for other problems in the year after dialysis initiation** |  |  |  | **0.04** |
| **0** | 957 (30%) | 1633 (29%) | 2590 (29%) |  |
| **1** | 826 (26%) | 1419 (25%) | 2245 (25%) |  |
| **≥ 2** | 1378 (44%) | 2643 (46%) | 4021 (45%) |  |
| **Hospital stays to prepare or maintain the vascular access** **in the year after dialysis initiation** |  |  |  | > 0.9 |
| **0** | 2069 (65%) | 3720 (65%) | 5789 (65%) |  |
| **≥ 1** | 1092 (34%) | 1975 (35%) | 2067 (35%) |  |
| **Hospital stays < 24h in the year after dialysis initiation** |  |  |  | **0.02** |
| **0** | 1485 (47%) | 2520 (44%) | 4005 (45%) |  |
| **1** | 666 (21%) | 1203 (21%) | 1869 (21%) |  |
| **≥ 2** | 1010 (32%) | 1972 (35%) | 2982 (34%) |  |
