# Supplementary material for: Care trajectory differences in women and men with end-stage renal disease after dialysis initiation
Source: PLoS One. 2023 Sep 14;18(9):e0289134. doi: 10.1371/journal.pone.0289134 (PMC10501619; doi:10.1371/journal.pone.0289134)
Supplement: S4 Table — (DOCX) [file pone.0289134.s004.docx]

## **S4 Table. Results of logistic regression model of number of hospital stays > 24h for other problem (>3 stays vs ≤ 3 stays) in the year after dialysis initiation (N=3,073)**

|  | **OR** | **95% CI** | **p-value** |
| --- | --- | --- | --- |
| **Sex** |  |  |  |
| **Women** | 1 | - | - |
| **Men** | 1.2 | [0.9 ; 1.4] | 0.14 |
| **Dialysis initiation and vascular access** |  |  |  |
| **Planned with fistula** | 1 | - | - |
| **Planned with catheter** | 1.3 | [1.01 ; 1.6] | **0.03** |
| **Emergency with fistula** | 1.6 | [1.03 ; 2.6] | **0.03** |
| **Emergency with catheter** | 1.4 | [1.1 ; 1.8] | **0.004** |
| **Albuminemia (g/L)** |  |  |  |
| **< 30** | 1 | - | - |
| **≥ 30** | 0.6 | [0.5 ; 0.8] | **< 0.001** |
| **Mobility** |  |  |  |
| **Total incapacity** | 1 | - | - |
| **Needs help** | 0.9 | [0.6 ; 1.3] | 0.5 |
| **Autonomous walking** | 0.7 | [0.4 ; 0.9] | **0.03** |
| **Treatment** |  |  |  |
| **Peritoneal dialysis** | 1 | - | **-** |
| **Hemodialysis** | 0.4 | [0.3 ; 0.7] | **0.001** |
| **Stand-alone dialysis** |  |  |  |
| **No** | 1 | - | - |
| **Yes** | 0.6 | [0.3 ; 0.9] | **0.04** |
| **Cirrhosis** |  |  |  |
| **No** | 1 | - | - |
| **Yes** | 1.8 | [1.2 ; 2.9] | **0.009** |
| **Active cancer** |  |  |  |
| **No** | 1 | - | **-** |
| **Yes** | 1.4 | [1.06 ; 1.7] | **0.01** |
| **Number of cardiovascular diseases** |  |  |  |
| **0** | 1 | - | **-** |
| **1** | 1.07 | [0.8 ; 1.4] | 0.6 |
| **2** | 1.2 | [0.9 ; 1.5] | 0.2 |
| **≥ 3** | 1.5 | [1.2 ; 1.9] | **0.001** |
| **Number of hospital stays >24h for other problems before dialysis** | 1.08 | [1.04 ; 1.1] | **< 0.001** |
| **Number of hospital stays >24h for kidney problems before dialysis** | 1.05 | [1.03 ; 1.1] | **< 0.001** |

OR, Odd Ratio; 95% CI, 95% Confidence Interval
