# Supplementary material for: Care trajectory differences in women and men with end-stage renal disease after dialysis initiation
Source: PLoS One. 2023 Sep 14;18(9):e0289134. doi: 10.1371/journal.pone.0289134 (PMC10501619; doi:10.1371/journal.pone.0289134)
Supplement: S5 Table — (DOCX) [file pone.0289134.s005.docx]

## **S5 Table. Logistic regression model of number of hospital stays < 24h (>4 stays vs ≤ 4 stays) in the year after dialysis initiation (N=4,073)**

|  | **OR** | **95% CI** | **p-value** |
| --- | --- | --- | --- |
| **Sex** |  |  |  |
| **Women** | 1 | - | - |
| **Men** | 1.1 | [0.9 ; 1.3] | 0.5 |
| **Dialysis initiation and vascular access** |  |  |  |
| **Planned with fistula** | 1 | - | - |
| **Planned with catheter** | 1.2 | [0.9 ; 1.6] | **0.05** |
| **Emergency with fistula** | 0.9 | [0.5 ; 1.4] | 0.6 |
| **Emergency with catheter** | 1.2 | [0.9 ; 1.5] | **0.07** |
| **Age (years)** |  |  |  |
| **18 – 45** | 1 | - | - |
| **45 – 60** | 0.7 | [0.5 ; 0.9] | **0.04** |
| **60 – 75** | 0.6 | [0.4 ; 0.7] | **< 0.001** |
| **> 75** | 0.4 | [0.3 ; 0.5] | **< 0.001** |
| **Type of nephropathy** |  |  |  |
| **Acute** | 1 | - | - |
| **Chronic** | 0.6 | [0.4 ; 0.7] | **< 0.001** |
| **Unknown** | 0.5 | [0.4 ; 0.7] | **< 0.001** |
| **Treatment** |  |  |  |
| **Peritoneal dialysis** | 1 | - | **-** |
| **Hemodialysis** | 0.2 | [0.1 ; 0.2] | **< 0.001** |
| **Active cancer** |  |  |  |
| **No** | 1 | - | - |
| **Yes** | 2.1 | [1.6 ; 2.7] | **< 0.001** |
| **Number of hospital stays < 24h before dialysis** | 1.03 | [1,01 ; 1.03] | **< 0.001** |
| **Number of GP consultations before dialysis** | 0.97 | [0.95 ; 0.99] | **0.001** |

OR, Odd Ratio; 95% CI, 95% Confidence Interval
