# Supplementary material for: Care trajectory differences in women and men with end-stage renal disease after dialysis initiation
Source: PLoS One. 2023 Sep 14;18(9):e0289134. doi: 10.1371/journal.pone.0289134 (PMC10501619; doi:10.1371/journal.pone.0289134)
Supplement: S6 Table — (DOCX) [file pone.0289134.s006.docx]

## **S6 Table. Logistic regression model of number of hospital stays to prepare or maintain the vascular access (1 stay vs 0 stay) in the year after dialysis initiation (N=4,182)**

|  | **OR** | **95% CI** | **p-value** |
| --- | --- | --- | --- |
| **Sex** |  |  |  |
| **Women** | 1 | - | - |
| **Men** | 1 | [0.8 ; 1.2] | 0.9 |
| **Dialysis initiation and vascular access** |  |  |  |
| **Planned with fistula** | 1 | - | **-** |
| **Planned with catheter** | 6.4 | [5.2 ; 7.8] | **< 0.001** |
| **Emergency with fistula** | 1.1 | [0.8 ; 1.7] | 0.5 |
| **Emergency with catheter** | 6.9 | [5.4 ; 8.7] | **< 0.001** |
| **Treatment** |  |  |  |
| **Peritoneal dialysis** | 1 | - | **-** |
| **Hemodialysis** | 0.5 | [0.4 ; 0.7] | **< 0.001** |
| **Number of cardiovascular diseases** |  |  |  |
| **0** | 1 | - | - |
| **1** | 1.1 | [0.9 ; 1.4] | 0.3 |
| **2** | 1.3 | [1.03 ; 1.7] | **0.02** |
| **≥ 3** | 1.0 | [0.8 ; 1.3] | 0.9 |

OR, Odd Ratio; 95% CI, 95% Confidence Interval
