# Supplementary material for: Care trajectory differences in women and men with end-stage renal disease after dialysis initiation
Source: PLoS One. 2023 Sep 14;18(9):e0289134. doi: 10.1371/journal.pone.0289134 (PMC10501619; doi:10.1371/journal.pone.0289134)
Supplement: S7 Table — (DOCX) [file pone.0289134.s007.docx]

## **S7 Table. Logistic regression model of number of hospital stays > 24h for kidney problem (1 stay vs 0 stay) in the year after dialysis initiation (N=5,869)**

|  | **OR** | **95% CI** | **p-value** |
| --- | --- | --- | --- |
| **Sex** |  |  |  |
| **Women** | 1 | - | - |
| **Men** | 0.8 | [0.7 ; 0.9] | **0.04** |
| **Dialysis initiation and vascular access** |  |  |  |
| **Planned with fistula** | 1 | - | **-** |
| **Planned with catheter** | 1.2 | [0.9 ; 1.5] | 0.2 |
| **Emergency with fistula** | 1.4 | [0.9 ; 2.2] | 0.1 |
| **Emergency with catheter** | 1.6 | [1.2 ; 2.1] | **< 0.001** |
| **BMI (kg/m²)** |  |  |  |
| **23 – 25** | 1 | - | - |
| **< 18.5** | 0.9 | [0.6 ; 1.6] | 0.8 |
| **18.5 – 23** | 0.8 | [0.6 ; 1.1] | 0.2 |
| **25 – 30** | 0.8 | [0.6 ; 1.05] | 0.1 |
| **≥ 30** | 0.7 | [0.5 ; 0.9] | **0.04** |
| **Mobility** |  |  |  |
| **Total incapacity** | 1 | - | - |
| **Needs help** | 0.9 | [0.5 ; 1.3] | 0.5 |
| **Autonomous walking** | 0.7 | [0.4 ; 0.9] | **0.04** |
| **Treatment** |  |  |  |
| **Peritoneal dialysis** | 1 | - | - |
| **Hemodialysis** | 0.4 | [0.3 ; 0.6] | **< 0.001** |
| **Number of hospital stays to prepare the vascular access before dialysis** | 0.7 | [0.6 ; 0.8] | **< 0.001** |
| **Number of hospital stays >24h for kidney problems before dialysis** | 1.04 | [1.01 ; 1.08] | **0.005** |

OR, Odd Ratio; 95% CI, 95% Confidence Interval
