# Supplementary material for: Care trajectory differences in women and men with end-stage renal disease after dialysis initiation
Source: PLoS One. 2023 Sep 14;18(9):e0289134. doi: 10.1371/journal.pone.0289134 (PMC10501619; doi:10.1371/journal.pone.0289134)
Supplement: S8 Table — (DOCX) [file pone.0289134.s008.docx]

## **S8 Table. Logistic regression model of number of GP consultations (>7 vs <=7) in the year after dialysis initiation (N=7,255)**

|  | **OR** | **95% CI** | **p-value** |
| --- | --- | --- | --- |
| **Sex** |  |  |  |
| **Women** | 1 | - | - |
| **Men** | 0.8 | [0.8 ; 0.9] | **0.04** |
| **Dialysis initiation and vascular access** |  |  |  |
| **Planned with fistula** | 1 | - | - |
| **Planned with catheter** | 1.1 | [0.9 ; 1.3] | 0.1 |
| **Emergency with fistula** | 0.9 | [0.6 ;1.2] | 0.4 |
| **Emergency with catheter** | 0.8 | [0.8 ; 1.0] | 0.06 |
| **Age (years)** |  |  |  |
| **18 – 45** | 1 | - | - |
| **45 – 60** | 1.2 | [0.9 ; 1.6] | 0.08 |
| **60 – 75** | 1.2 | [0.9 ; 1.5] | 0.09 |
| **> 75** | 1.6 | [1.3 ; 1.9] | **< 0.001** |
| **Mobility** |  |  |  |
| **Total incapacity** | 1 | - | - |
| **Needs help** | 0.9 | [0.7 ; 1.3] | 0.8 |
| **Autonomous walking** | 0.6 | [0.4 ; 0.8] | **< 0.001** |
| **Diabetes** |  |  |  |
| **No** | 1 | - | **-** |
| **Yes** | 1.3 | [1.2 ; 1.5] | **< 0.001** |
| **Number of cardiovascular diseases** |  |  |  |
| **0** | 1 | - | **-** |
| **1** | 1.1 | [0.9 ; 1.3] | 0.06 |
| **2** | 1.1 | [0.9 ; 1.3] | 0.3 |
| **≥ 3** | 1.05 | [1.2 ; 1.7] | **< 0.001** |
| **Number of hospital stays >24h for kidney problems before dialysis** | 1.05 | [1.01 ; 1.08] | **0.001** |

OR, Odd Ratio; 95% CI, 95% Confidence Interval
