# Supplementary material for: Care trajectory differences in women and men with end-stage renal disease after dialysis initiation
Source: PLoS One. 2023 Sep 14;18(9):e0289134. doi: 10.1371/journal.pone.0289134 (PMC10501619; doi:10.1371/journal.pone.0289134)
Supplement: S9 Table — (DOCX) [file pone.0289134.s009.docx]

**S9 Table: Number of hospital stays for men and women by hospital type (N=42,106)**

|  | **Women**  **N=14299**  **Number (%)** | **Men**  **N=27807**  **Number (%)** | **p-value**  **(Chi^2^ test)** |
| --- | --- | --- | --- |
| **Hospital type** |  |  | **< 0.001** |
| **Public-sector hospitals** | 8753 (61%) | 17948 (64%) |  |
| **Private non-for profit clinics** | 1069 (8%) | 2187 (8%) |  |
| **Private for profit clinics** | 4471 (31%) | 7671 (28%) |  |
